# Increasing the Impact of Impact Evaluation

**DOI:** 10.4269/ajtmh.17-0600

**Published:** 2017-09-27

**Authors:** Richard E. Cibulskis

**Affiliations:** Global Malaria Programme, World Health Organization, Geneva, Switzerland

Evaluation of public health programs is critical for ensuring the accountability of resources, for learning, and ultimately, for program improvement. The evaluation of malaria programs poses specific challenges. First, malaria predominantly occurs in countries and areas with relatively weak information systems. Second, a key outcome measure—deaths caused by malaria—is not easily measured. Third, transmission of malaria is highly influenced by fluctuations in rainfall and temperature, which can prompt large increases or decreases in case incidence that disguise the effects of malaria interventions. Fourth, changes in malaria program coverage may be associated with changes in other interventions and in socioeconomic conditions.

The articles included in this supplement describe advances in how evaluations can be conducted in resource-poor settings and how links can be established between malaria interventions and health outcomes while disentangling potential confounders. They exploit a range of methodological techniques (establishing a plausibility argument, dose-response analyses, interrupted time series analyses, and retrospective cohort analysis) and data sources (household survey data, routine health management information systems, and climate data). By using multiple approaches and data sources, the articles add to the body of evidence demonstrating that malaria interventions have contributed to substantial reductions in malaria and in childhood mortality; the wide-scale deployment of malaria interventions has led to a world very different from that of 15 years ago.

However, although huge advances have been made in extending malaria program coverage since the beginning of the millennium, millions of people living in malaria endemic areas still do not have access to malaria interventions, and the financing of malaria programs has fallen short of targets.^1^ In the presence of market failures, evaluation plays an important role in the health sector as a mechanism for ensuring that appropriate levels of investment are made in different programs. Malaria programs have benefited enormously from international investments, which comprised approximately 50% of malaria program financing in 2015.^1^ Evidence that malaria interventions have had a substantial health impact will be fundamental to ensuring continued or expanded contributions from international donors, though the scope for expansion of international assistance appears limited.^2,3^ It is therefore critical that the results of malaria evaluations are not only targeted to international donors, but that they are also used in national health and development planning in malaria endemic countries and incorporated into the budget process. For this to occur, strategies for conducting evaluations may need to be adapted to make them more locally relevant. Although a focus on health impact is still important—and persuasive—greater emphasis may need to be given to analyzing who benefits from interventions in a country and who does not, the costs of interventions, and what aspects of programs need to be strengthened; the linking of health impact evaluations with national malaria program reviews would be beneficial.

Moreover, although periodic formal evaluations are necessary, they are not sufficient to ensure efficient resource allocation and program implementation. Strengthening of continuous monitoring and surveillance systems and national capacity is fundamental to ensuring resources are directed to the most affected populations, that gaps in program coverage are identified, and that disease outbreaks are detected.^4^ Accordingly, the World Health Organization *Global Technical Strategy for Malaria 2016–2030* highlights the need for malaria surveillance to be transformed into a core intervention, recognizing that the enhanced use of information can itself act as a powerful intervention and in this case further accelerate declines in malaria.
